# Biases in bioethics: a narrative review

**DOI:** 10.1186/s12910-023-00894-0

**Published:** 2023-03-06

**Authors:** Bjørn Hofmann

**Affiliations:** 1 Institute for the Health Sciences at the Norwegian University of Science and Technology (NTNU), PO Box 191, 2801 Gjøvik, Norway; 2 The Centre of Medical Ethics at the University of Oslo, Oslo, Norway

**Keywords:** Bias, Imperative, Cognitive, Affective, Quality, Ethics, Distortion, Judgment

## Abstract

Given that biases can distort bioethics work, it has received surprisingly little and fragmented attention compared to in other fields of research. This article provides an overview of potentially relevant biases in bioethics, such as cognitive biases, affective biases, imperatives, and moral biases. Special attention is given to moral biases, which are discussed in terms of (1) Framings, (2) Moral theory bias, (3) Analysis bias, (4) Argumentation bias, and (5) Decision bias. While the overview is not exhaustive and the taxonomy by no means is absolute, it provides initial guidance with respect to assessing the relevance of various biases for specific kinds of bioethics work. One reason why we should identify and address biases in bioethics is that it can help us assess and improve the quality of bioethics work.

## Background

Biases can broadly be defined as “pervasive simplifications or distortions in judgment and reasoning that systematically affect human decision making” [1]. There is an extensive literature on biases in general, and a wide range of biases have been identified for specific professions, including health professionals and health researchers [2–8].

In psychology and behavioral economics, more than 180 biases have been identified, and in empirical research there are many measures to estimate, avoid, and reduce biases [9–11]. Compared to other fields, biases have been gained less attention in bioethics.

Certainly, biases have been mentioned in bioethics. For example, some biases have been identified in clinical ethics committees work [12], and specific biases have been recognized, such as “whiteness in bioethics” [13, 14], gender bias (“maleness”) [15, 16], western bias [17], cultural bias [18], and geographical bias (in terms of which topics that are discussed in bioethics) [19]. Specific biases have also been shown to undermine patient autonomy [20, 21] and health professionals’ autonomy [22]. Bias amongst journal editors [23] may be relevant for bioethics, and biases have been identified in specific practices, such as in nursing ethics and health care research ethics [24–26]. Moreover, outcome bias have been demonstrated in post hoc ethical judgment as “the same behaviors produce more ethical condemnation when they happen to produce bad rather than good outcomes, even if the outcomes are determined by chance”[27].

Nonetheless, compared to other areas, biases have attracted surprisingly little and fragmented attention in bioethics. Does this mean that there are no biases in bioethics and its literature? Or does it mean that there are biases in bioethics, but we have no need to identify [28] or avoid them? Or is the reason that we do not have any means to avoid them, making it unnecessary to address them? Or that we are dealing with them under other names? These are relevant question to be addressed in this article.

Accordingly, the key question for this article is: *what kind of biases can be relevant in bioethics, and what can we do about them*? While it is impossible to provide an exhaustive list of and in-depth analysis of all biases in bioethics, this article tries to provide an overview and to suggest a classification of biases *in* bioethics. This will be done by asking three specific questions:

1. What is the relevance for bioethics of various biases identified in other fields of research?
2. For which type of bioethics work are these biases (more or less) relevant?
3. Are there biases specific to bioethics work?

Given the vast number of biases in the literature it is impossible to provide a detailed description and deep analysis of all of them. For that purpose, I refer the reader to the special literature (found in the references). The goal of this article is more modestly (a) to provide a compilation of a wide range of research to facilitate the access to a fascinating and important field to newcomers (i.e., an educational purpose), (b) to demonstrate an approach to investigate biases in bioethics in a systematic manner (typology), and (c) to draw attention to a topic that deserves more explicit attention than it has obtained (i.e., to stimulate debate). However, the overall aim is to contribute to the improvement of bioethics, as biases are something that can distort or reduce the quality of our work, and the first step is to identify and to acknowledge them.

### Some initial distinctions and clarifications

The provided definition of bias is very broad: “pervasive simplifications or distortions in judgment and reasoning that systematically affect human decision making” [1]. It can be argued that it is directed towards rapid decision-making and not more elaborate reflection [9, 11, 29]. This is true, but the task in this article is to investigate the relevance for bioethics. Hence, “decision making” must reflect the decisions that we do in bioethics.

Moreover, biases have been defined in the field of ethics as “faulty beliefs, attitudes, or behavioral tendencies that constrain cognition and thereby inhibit an individual's ability to make ethical decisions” [30]. While the latter definition works well, this article will not be constricted to “cognition” in a narrow sense, but includes affection, judgment, and moral deliberation.

Additionally, it can be relevant to differentiate between bias in the process, in the content (of the outcome of ethics work), and in the characteristics of (specific) bioethicists. Clearly, as bioethicists we are subject to a wide range of biases and prejudices in all parts of our activities. Due to limited space, this article will focus more specifically on the biases that can appear in our activities, i.e., in doing bioethics.

As indicated, biases may pose ethical problems, such as stigmatization, discrimination, and injustice. As with psychological biases, biases in bioethics may result in unsafe, ineffective, or unwarranted care [31]. While highly important and ethically relevant, the consequences of biases in actual care are beyond the topic of this study and will be left for other and more specific investigations.

Accordingly, it can be helpful to differentiate between various types of biases depending on the type of bioethics work. Bioethics is a type of applied ethics [32] that has been defined as “a field spanning a range of different philosophical approaches, normative standpoints, methods and styles of analysis, metaphysics, and ontologies” [33]. While there are many ways to classify the activities within bioethics, the following appears relevant for this study:

- *Philosophical, ethical, and conceptual analyzes (PEC)*: theoretical or explorative analysis without explicit normative conclusion, decision, favoritism, or advice.
- *Ethical analyzes (EA)* in which bioethicists explores the evidence and relevant argumentation, provide careful deliberation, and provide a well-founded conclusion (working as a judge in the terminology of Haidt [34]).
- *Clinical ethics consultation (CEC)*: Communication and facilitating decision-making [35].
- *Agitation (A)*: where bioethicists argue (one-sidedly, rhetorically, or defensive) for a given view (and function as lawyers in the wording of Jonathan Haidt [34]).
- *Empirical research (ER)*: in which bioethicists contribute to producing empirical evidence on normative issues.
- *Ethics literature synthesis (ELS)*: Summaries of normative literature, such as in reports etc. [36].

Moreover, there may be differences between biases in meta-ethics, normative ethics, empirical, applied, and descriptive/empirical ethics [32]. Although the biases that are discussed in this article are identified in bioethics, they may be relevant for other types of as well.

In the following I will provide an overview of and address biases in bioethics work and introduce a taxonomy that hopefully can be useful for (a) educational and (b) classificatory purposes, as well as (c) stimulate debate and further research. Please note that the taxonomy is neither exhaustive nor absolute.

## Methods

In order to identify biases in bioethics, a narrative (overview) review [37] was conducted. An initial search was performed in PubMed (February 5 2022) with the search string ((bias[MeSH Terms]) AND ((bioethics[MeSH Terms]) OR (medical ethics[MeSH Terms])).

156 references were identified and screened for titles and abstracts. References that thematically did not address ethical issues or were not related to biases relevant for bioethics were excluded. Of the 43 full-text examined papers, 32 were included. Based on snowballing techniques, 74 references were added from the included papers and further 61 relevant articles referred to in these were included.

Additionally, based on previous research [7, 38], biases in psychology and behavioral economics were reviewed and those relevant for bioethics were included. References for and examples of these were identified by specific (targeted) searches. As the goal was to find relevant examples (and not to be exhaustive), only one or two examples or references were included.

## Main text

### Mental Biases

In accordance with the extensive studies of biases in psychology and behavioral economics it is reasonable to differentiate between cognitive and affective biases, as they can distort and bias bioethics work. Additionally, I have identified imperatives (as a type of bias) and moral biases (see below). The space does not allow for mentioning, explaining, and exemplifying of all the biases that are relevant in bioethics. While several biases will be listed, defined, and explained in tables, only some will be discussed due to the very many biases and limited space. Moreover, biases in the bioethics literature are identified to come in three kinds: (1) the description of biases in the field of interest described in articles on bioethical issues, e.g., how health professionals may have specific cognitive biases in clinical decision-making; (2) biases as explanations of positions or arguments in bioethics, for example that the withholding-withdrawal-distinction is a result of loss aversion; (3) biases in doing bioethics (e.g., in arguments or reasoning). As the latter two are the most important for the quality of bioethics work, the emphasis will be on those.

### Cognitive biases

The psychology and behavioral economics literature have identified a wide range of cognitive biases [9, 11, 29]. Many of these are relevant for bioethics, as they influence the cognitive aspects of ethical judgments and decision making [30, 39]. Table 1 provides a selection of cognitive biases that can be relevant in bioethics and are worth considering, a definition and/or short description of these biases as well as in which type of bioethics work the bias mainly relates to.

**Table 1:** Cognitive biases relevant for bioethics listed in alphabetical order with explanations and an indication for what type of bioethics this bias may be most relevant to assess

Cognitive bias	Definition/short description	Type of bioethics
Ambiguity effect	A tendency to avoid options for which the probability of favorable outcomes is unknown	A, (ER), EA, CEC, PEC
Anchoring effect	Initial information is overrated, ignoring high quality evidence that can be more difficult to obtain	A, ER, EA, ELS, CEC, PEC
Apophenia	The tendency to perceive connections between things, events, or perspectives that are unrelated	A, ER, EA, CEC
Availability bias	Overestimating the likelihood of (recent) events easily available in memory or how unusual or emotionally charged they are	A, ER, EA, CEC
Confirmation bias	The tendency to focus on information in a way that confirms one's preconceptions or expectations. Related to the tendency to reject new evidence that contradicts a perspective or paradigm (*the Semmelweis reflex*)	A, ER, EA, ELS, CEC, PEC
Default effect	The tendency to favor the default option when given a choice between several options	A, ER, EA, ELS, CEC, PEC
Endowment effect	Overvaluing what we already have got compared to alternatives, as it is more difficult to give up than to acquire something (such as argument, perspective, line of reasoning)	A, ER, EA, ELS, CEC, PEC
Extension bias	The tendency to think that more is better than less. This is related to the conflation of quality with quantity	A, EA, CEC
False priors (stereotyping)	Presuming that members of a group have specific characteristics without having information about them. Gender bias is an example of false priors	A, ER, EA, ELS, CEC, PEC
Familiarity bias	The tendency to express unwarranted liking for events, items, theories, or perspectives merely because of familiarity with them	A, ER, EA, ELS, CEC, PEC
Focusing illusion	Too much focus on certain details, ignoring other relevant factors	A, ER, EA, ELS, CEC, PEC
Framing (information) effect	Drawing conclusions from information depending on how the information is presented	ER, ELS, PEC
Hard–easy effect	The tendency to overestimate the ability to accomplish hard tasks, as well as underestimate the ability to accomplish easy tasks	A, ER, EA, ELS, CEC, PEC
Hindsight bias	The inclination to see past events as being predictable at the time those events happened. Related to *outcome bias* where decisions are assessed by its outcomes rather than by the quality of the decision at the time of making	EA, PEC
Illusion of control	The tendency to overestimate the degree of influence over external events	A, (EA), CEC
Illusion of validity	The tendency to overestimate the accuracy of one's judgments, especially when available information is coherent or consistent	A, ER, EA, ELS, CEC, PEC
Implicit bias	The tendency that underlying attitudes and stereotypes are attributed to persons or groups of people that affect how they understand, judge, and engage with them. Also called *unconscious bias*	A, ER, EA, CEC, PEC
Information bias	The tendency to seek information even when it cannot affect the decision	ER, ELS, PEC
Law of the instrument	The tendency to rely on familiar methods, ignoring or devaluing alternative approaches	A, EA, CEC, PEC
Normalcy bias	The reluctance to take into account, or plan for, or react to, a severe event (e.g., a disaster) which has not happened before or very long ago (such as a pandemic)	A, ER, EA, ELS, CEC, PEC
Ostrich effect	The tendency to ignore an obvious (negative) situation, perspective, or alternative	A, EA, CEC, PEC
Overconfidence effect	The tendency of having excessive confidence in one's own judgments, answers, and decisions	A, EA, CEC, PEC
Present bias	The tendency for to have a stronger preference for more immediate issues, outcomes, or solutions compared to more long term issues, outcomes, or solutions	A, ER, EA, ELS, CEC, PEC
Probability neglect	The tendency to neglect probabilities when making decisions under uncertainty. Small risks may be totally neglected or vastly overrated	A, ER, EA, ELS, CEC, PEC
Pro-innovation bias	The tendency to have an excessive optimism towards innovations (ignoring limitations and weaknesses)	A, ER, EA, ELS, CEC, PEC
Projection bias	The tendency to overestimate how much future situations will assemble present situations leading to sub-optimal decisions (when not correct). This is related to *impact bias*	A, ER, EA, CEC, PEC
Proportionality bias	The tendency to assume that big events have big causes and that important events have important reasons	A, EA, CEC, PEC
Rhyme as reason effect	The tendency to think that rhyming or catchy statements or arguments are perceived as more truthful or sound	A, EA, PEC
Synecdoche effect	One specific characteristic comes to signify the whole person	A, ER, EA, ELS, CEC, PEC
Truthiness bias	A tendency to believe that a statement is true due to the strength of one’s belief in the conclusion (belief bias, subjective validation bias) or how easy it is to process or how many times it is stated (the illusory truth effect)	A, ER, EA, CEC

*A* Agitation, *ER* Empirical Research, *EA* Ethical analyzes, *ELS* Ethics literature synthesis, *CEC* Clinical ethics consultation, *PEC* Philosophical, Ethical, and Conceptual analyzes

One bias that can be observed in the bioethics literature is the *extension bias*. For example, it is frequently thought that more blood tests and radiological examinations are better than few [40]. Correspondingly, in the enhancement debate it has been argued that more intelligence is better than little or normal intelligence [41]. Moreover, such cognitive biases are relevant in the ethics of priority setting where providing many low-value services erroneously is considered to be of great value [6]. Moreover, we sometimes tend to think that the more arguments we can find for a decision, the better (neglecting quality). Hence, the general tendency to think that more is better than less appears to have some relevance in bioethics as well.

According to the so-called *focusing illusion* we tend to focus too much on certain details, ignoring other factors [42]. This bias is particularly relevant in complex cases where we may come to base ethical analyses on specific aspects and premises (such as facts, values, principles). Moreover, it may be relevant for ethical arguments, where we can come to focus solely on specific principles, e.g., on the principle of personal autonomy in assessing prenatal screening [43]. The focusing illusion is related to the *prominence effect* (see below) and the *anchoring effect*, where we tend to rely too much on initial information, and ignore high quality evidence (or context information) that may be more difficult to obtain [8].

*Confirmation bias* is the tendency to focus on information in a way that confirms one's preconceptions or expectations and is related to what has been called the “*self-serving bias*,” i.e., the tendency unwittingly to assimilate evidence in a way that favors a conclusion that we stand to gain from [44]. Confirmation bias may not be restricted to evidence, but may also include intuitions, arguments, and judgments that support a specific bioethical perspective or conclusion.

Another bias worth paying careful attention to is the *endowment effect* according to which we can come to overvalue what we already have got (or obtained) compared to alternatives. While bioethicists do not obtain or depend on things (as in experiments on the endowment effect), the same psychological mechanism may be relevant for our relationship with argument, perspectives, lines of reasoning, theoretical positions etc. In the same way that we tend to demand much more to give up an object than we would be willing to pay to acquire it [45], we may cling to a specific perspective or position in bioethics. Once we have an insight or a view, we may not be willing to give it a way or replace it by another, even if it may be better. As such, an “endowment effect” in bioethics can spur conservativism [46].

The tendency to overestimate the accuracy of one's own’s judgments, i.e., *the illusion of validity*, appears to be as relevant in bioethics work as elsewhere [47]. The same goes for the tendency to rely on familiar methods, ignoring or devaluing alternative approaches [48]. Bioethicists narrowly following one approach, be it (rule-)utilitarianism or deontology, are subject to the *law of the instrument*.

Other general biases may be relevant in bioethics work as well, such as the *implicit bias* which is described as the tendency to let underlying attitudes and stereotypes attributed to persons or groups of people affect how we understand, judge, and engage with them (without being aware of it) [49]. This has also been labelled “unconscious bias” and is related to the *synecdoche effect*, where one specific characteristic comes to signify the whole person [50], e.g., where persons with certain disabilities are addressed in terms of their disability, and not as a person.

Also, in bioethics we may be subject to *present bias*, e.g., when we show a stronger preference for addressing more immediate issues, outcomes, or solutions compared to more long-term problems, outcomes, or solutions. When we face with topical cases in the clinic or in the media and are expected to suggest solutions, more long-term and principled issues may be overshadowed [51, 52].

*Probability neglect* is the tendency to neglect probabilities when making decisions under uncertainty. This seems to be a general psychological bias that may be relevant when we assess potential outcomes of decisions or actions [53, 54]. Empirical premises are crucial to many types of bioethics work, and we may come to neglect small risks or come to totally overrate them. For example, bioethicists arguing for germline gene editing (GGE) may downplay off-target effects: “it is plausible that as GGE develops the rate of off-target mutations will become negligible. The rates of off-targets mutations in animal models have been declining rapidly, and such mutations are now considered ‘undetectable’ in some applications” [55]. Others may overrate such effects.

The tendency to have an excessive optimism towards innovations (*pro-innovation bias*) is also known in healthcare [56, 57], where some bioethicists are known to be very positive to specific innovations, such as CRISPR-cas9, and others are optimistic with respect to technological innovations in general [58]. The problem is that they may ignore limitations and weaknesses. The opposite is also true, of course. See status quo bias below.

The relevance of the *rhyme and reason effect* can be illustrated with John Harris’ elegant argument:”I have a rational preference to remain nondisabled, and I have that preference for any children I may have. To have a rational preference not to be disabled is not the same as having a rational preference for the nondisabled as persons” [59]. While catchy, it is not clear that the claim holds [60].

*Implicit biases* “involve associations outside conscious awareness that lead to a negative evaluation of a person on the basis of irrelevant characteristics such as race or gender” and are prevalent amongst health professionals [2] and bioethicists [61, 62]. Because they can operate to the disadvantage of those who are already vulnerable such biases are relevant in bioethics. Examples include minority ethnic populations, immigrants, poor people, low health-literacy individuals, sexual minorities, children, women, the elderly, the mentally ill, persons with overweight and disability. Even more, anyone may be rendered vulnerable given a certain context [63].

Common to all the cognitive biases is that they may distort our reasoning in bioethics. Moreover, while all are relevant in bioethics, they may be more or less relevant in different types of bioethics work as indicated in the table.

### Affective biases

While the distinction between cognitive and affective biases is debatable, several scholars prefer to differentiate between them. The readers who dispute this distinction, can add the following to the cognitive biases above. Table 2 provides a brief overview of the affective biases with definitions/descriptions and indications of which type of bioethics work the bias mainly relates to. Then follows a brief discussion of some of the biases.

**Table 2:** Affective biases relevant for bioethics listed in alphabetical order with explanations and an indication for what type of bioethics this bias may be most relevant to assess

Affective bias	Definition/short description	Type of bioethics
Affective forecasting	The tendency to let present emotional states and conceptions to be projected to future events	A, EA, CEC
Aversion to risk, uncertainty, or ambiguity	The tendency to let the fear of risk, uncertainty, or ambiguity influence decision-making	A, EA, (ELS), CEC
Compassion fade	The tendency to be more compassionate towards few identifiable victims than towards many anonymous ones, which is related to identifiability bias (bias towards caring more for the identified)	A, EA, CEC
Empathy gap	The tendency to underestimate the influence or strength of feelings, in either oneself or others	A, EA, CEC
Exaggerated expectation	The tendency to expect or predict more extreme outcomes than those that actually happen	A, EA, CEC, PEC
Identifiability bias	The inclination to focus on identified persons (or issues) and give them priority. Related to the “singularity effect”	A, EA, ER, CEC
Impact bias	The tendency to overestimate the impact of a future event	A, EA, CEC
Loss aversion	The perceived disadvantage of giving up an item is greater than the utility associated with acquiring it	A, EA, CEC
Omission bias	The tendency to judge harmful actions (commissions) as worse than equally harmful inactions (omissions)	A, EA,d, CEC
Optimism bias	The tendency to overestimating the probability and beneficence of favorable outcomes and to those of underestimate of undesirable outcomes (this is related to other biases, such as *wishful thinking*, the *valence effect*, *positive outcome bias*, and *pro-innovation bias*)	A, EA, CEC
Pessimism bias	The tendency to overestimate the probability and negative consequences of negative outcomes or things happening	A, EA, CEC
Projection bias	The inclination to think that others have the same attitude, belief, or priority that one has oneself, even when this is unlikely	A, EA, CEC
Prominence effect	The tendency that one dominant factor determines the decision-makers’ preferences. This relates to what has been called the *scope neglect*, *scope insensitivity*, and *opportunity cost neglect*	A, ER, EA, ELS, CEC, PEC
Pseudo-certainty effect	The tendency to make risk-averse decisions for positive expected outcomes and risk-seeking for expected negative outcomes	A, EA, CEC
Salience bias	The tendency to focus on items that are emotionally more prominent or striking and ignore those that are less so	A, EA, CEC
Yuck factor	The tendency to react to events, things, persons, or groups of persons based on disgust	A, ER, EA, ELS, CEC, PEC

*A* Agitation, *ER* Empirical Research, *EA* Ethical analyzes, *ELS* Ethics literature synthesis, *CEC* Clinical ethics consultation, *PEC* Philosophical, Ethical, and Conceptual analyzes

In bioethics individual cases can be paradigmatic, such as the cases of Karen Ann Quinlan, Terri Shiavo, and Charlie Gard. However, identified individuals, conditions, or groups of persons can induce special sympathy and empathy (or the opposite). This can engender unwarranted attention and priority towards specific groups in bioethics due to what has been called the *identifiability bias* and “the singularity effect” [64] and “bias towards identified victims,” [65] but also to “compassion fade” [66, 67]. Hence, identification can induce importance in biased manners.

*Affective forecasting* is another type of bias where one’s emotional state and conception is projected to future events [68]. Examples from bioethics is cases where hopes and desires flavor the ethical assessment of emerging technologies [7]. Related to affective forecasting is the *impact bias*, which is the tendency to overestimate the impact of a future event [69]. It can be observed in bioethics debates on novel technologies where future benefits are taken for granted, e.g., on gene editing, personalized/precision medicine, BigData, and Artificial Intelligence, and relates to *projection bias* (see Table 2).

On the other hand, as bioethicists we may let aversions to dangers or uncertainties influence our work and be subject to biases such as *aversion to risk* [70, 71] and *aversion to ambiguity* [8]. These biases may make bioethicists promote excessive diagnostics (overdiagnosis) and therapeutics (overtreatment) [6].

*Loss aversion* means that the perceived disadvantage of giving up an item is greater than the utility associated with acquiring it [72]. As with the endowment effect (and others) bioethicists do not obtain things or items. Nonetheless, the same psychological mechanism may be relevant for our relationship with argument, perspectives, lines of reasoning, and theoretical positions. We have invested many years of studies, research, and work experience in specific approaches or positions, and leaving them could result in the same affective effects. On the other hand, the bias of loss aversion has been applied to explain (or undermine) a bioethical argument, such as the difference between withholding and withdrawing treatment [73]. Hence, the same bias may be used in explanation or argumentation as being relevant for us as bioethicists.

While bioethics address complicated and complex issues, we may come to simplify and let a few or even one dominant factor determine our final analyses, which resembles what is defined as *the prominence effect*, which relates to what has been called “the scope neglect,” “scope insensitivity,” and “opportunity cost neglect.” The point is that we lose important aspects by narrowing our scope.

The yuck factor has been extensively discussed in the ethics literature [74–77] and there are different conceptions of whether it directs or distorts moral reasoning. The point here is not to provide the final answer to this question, but more modestly to point out that it can influence reasoning in bioethics in covert manners.

Hence, the affective biases may distort our reasoning in bioethics as do cognitive biases. Accordingly, being able to identify them is the first step to addressing and handling them—and thereby to improve bioethics.

### Imperatives

Another type of distortion of judgments are imperatives, which are also oftentimes called biases. Imperatives are actions that are felt needed despite dim outcomes. They are immediate reflections of long-established doctrine or belief, and can be rooted in deontology [78]. Status quo bias [46] and progress bias [58] are but two examples presented in Table 3.

**Table 3:** Imperatives relevant for bioethics listed in alphabetical order with explanations and an indication for what type of bioethics this bias may be most relevant to assess

Imperative	Explanation	Type of bioethics
Status quo bias	The tendency to like things to stay relatively the same. Also related to *system justification*	A, ER, EA, ELS, CEC, PEC
Imperative of Knowledge	The tendency to think that it is better to know than not to know	A, EA, ELS, CEC
Imperative of the primus	The tendency to think that early action or decision-making is better than late	A, EA, CEC
Competency effect	The tendency to think that formal competency will result in better decision-making	A, EA, CEC, PEC
Progress bias	The tendency to argue for specific solutions because they symbolize novelty and progress. This is related to what has been called *adoption addiction*	A, ER, EA, CEC
Complexity bias	The tendency to think that advanced systems or solutions are better than simple	A, EA

*A* Agitation, *ER* Empirical Research, *EA* Ethical analyzes, *ELS* Ethics literature synthesis, *CEC* Clinical ethics consultation, *PEC* Philosophical, Ethical, and Conceptual analyzes

*Status quo bias* is described as an irrational preference for an option only because it preserves the current state of affairs [79]. This may result from people’s *aversion to change* (conservativism), making them avoiding changing practice [8], or system justification, i.e., the need to have stability in bioethical theory and practice, even despite these being dysfunctional or hampering improvements [80]. The *Status quo bias* is also associated with the cognitive bias called the *endowment effect*, according to which we tend to overvalue what we already have got compared to alternatives. See above.

Contrary to the status quo bias there also is a *progress bias*, according to which persons experience a strong propensity to promote what is considered to be progressive [58]. It is also related to what has been called *pro-innovation bias* and *optimism bias* (see above). Additionally, progress bias is related to what has been called *adoption addiction*, according to which we appear to have a tendency to be more interested in assessing and investing in new and shiny technologies than reassessing and disinvesting in old and inefficient technologies [81]. In bioethics status quo bias and progress bias are particularly relevant with respect to the assessment of biotechnologies [58].

In ethical debates on genomic analysis, incidental findings, return of results, newborn screening, and prenatal screening we often encounter the argument that people have the right to know [82] or that not providing a test (or its results) is denying them crucial information [83]. Certainly, holding back information can undermine respect for autonomy, but this is not always the case in the mentioned examples [84]. This indicates that *the imperative of knowledge* is relevant in bioethics debates as it is in healthcare and society in general [7].

Correspondingly, there may be a *competency effect* in bioethics, as there is a tendency to think that ethicists with better formal competency will produce better bioethics work which results in better decision-making. Again, this may be the case, but it is certainly not always so. Prominent bioethicists may be extremely busy not having enough time to apply the full capacity of their competency for all purposes.

Again, these (and other imperatives) [7] may influence, undermine the quality, and even distort our work in bioethics.

So far, this review illustrates that a wide range of cognitive and affective biases as well as imperatives are relevant in bioethics work. Clearly, as general mental mechanisms they influence bioethicists in the same manner as the general population. What I have tried to investigate in addition is whether the same psychological mechanisms may have any particular relevance to bioethics work. In addition to the “mental mechanisms” identified above, the literature reveals that there are “moral mechanisms” that can (negatively) influence bioethics work.

### Moral bias

In the same manner as our thoughts, affections, and imperatives may influence or even distort our moral judgments, so may various moral mechanisms. Our moral judgments may be influenced by our ethical positions, religious beliefs, methodological preferences, and moral inclinations. Accordingly, *moral bias can be defined as moral beliefs, attitudes, perspectives, or behavioral tendencies that unwittingly tend to influence our moral judgment in specific directions*.

Again, space does not allow for an exhaustive review of all kinds of moral bias. Only biases that can be important to acknowledge and address and that can contribute to improve the quality of bioethics work are included. To facilitate reading, the biases are grouped in five groups: (1) Framings, (2) Moral theory bias, (3) Analysis bias, (4) Argumentation bias, and (5) Decision bias. The table at the end provides a summary of the biases and indicates for what type of bioethics they may be most relevant. Please note that the groupings are not absolute.

#### Framings

A moral framing effect can be defined as a bias where people’s moral judgments are influenced by how the options or arguments are framed or by the (ethical) framings of moral situations or challenges.

One type of moral bias that can be understood as a framing is *standpoint adherence*, according to which we are not willing to change standpoint despite solid evidence. Empirical research shows that strong positions are difficult to change, even with good evidence [85]. This relates to cognitive biases such as the *ostrich effect* and *overconfidence effect*. Bioethicists that change their standpoint, method, or perspective are rarely heard of. However, it is worth noting that some experiments have shown that some of our preferences are easy to influence (*choice blindness* and *preference change*) [86].

Moreover, there may be framing effects in the terminology we apply. Although bioethicists have increasingly become aware of the normative relevant difference between “epileptics” and “persons with epilepsy,” we have used terms such as “hypochondriacs,” “diabetics,” and “downs children” etc. [87]. The moral relevance of this has been discussed in relation to the synecdoche effect [50] (discussed above).

One reason for the terminology problem may result from how bioethicists are personally, socially, and culturally embedded: “bioethics is an embedded socio-cultural practice, shaped by the everchanging intuitions of individual philosophers, and cannot be viewed as an intellectual endeavour detached from the particular issues that give rise to, and motivate, that analysis”[88].

Corresponding to what in business ethics research has been called the *social desirability response bias* [89] and what in the Science and Technology Studies (STS) literature has been coined *tacit commitments* and *narrative bias* [90] there can be an *expectation bias* in bioethics making the social expectations influence the bioethics work. In clinical ethics several such biases have been identified in terms of “bias towards the interests of hospital management,” “bias towards laws and regulations,” “bias towards individuals’ perspectives and interests,” and “bias towards the perspectives and interests of health-care professions”[12]. Such biases stem from conflicts of interest and seem especially relevant when bioethicists work in or for expert groups. Thus, *expectation bias* is related to social mechanisms and motivations.

As illustrated by the research of Don A. Moore and colleagues, self-interest tend to operate via automatic processes in *conflict of interests* [91–93] and Mahdi Kafaee and colleagues have experimentally demonstrated how conflict of interest might shape the perception of a situation in a subconscious manner [94]. Consequently, conflicts of interest can (unconsciously) bias bioethics work [95]. Clearly, ethicists are hired by stakeholders, and can have conflict of interests as other researchers [96, 97], especially in settings where bioethics has become a business [98]. Moreover, there may be professional conflicts of interest, e.g., between ethicists and jurists or policy makers [99, 100]. Ethicists may also have strong opinions in controversial issues biasing their judgments [101] or be subject to political attention (“political bias”) [102]. As acknowledged, conflicts of interests have been identified as biases in clinical ethics committee work [103].

According to the *impartiality illusion*, we may think that we are impartial while closer analysis (by independent and blinded reviewers) may reveal specific tendencies, inclinations, or partiality. Everett provides one interesting example about endorsing consequentialism [104].

Another well-known way to frame a bioethical debate is by defining what is (not) the issue (“that’s not the question”) and identifying what is an ethical problem [105]. Such delimiting claims seem to be common [106–111] and can easily result in biased bioethics.

Hence, there may many types of unconscious framings that direct bioethics work. Being aware of and addressing these framings may contribute to improve bioethics work.

#### Moral theory bias

There are also biases with respect to moral theories, i.e., where moral theories may direct specific moral challenges are perceived, defined, deliberated, and solved. One such bias is the *theory dominance* according to which one theoretical perspective inherently dominates the analysis, ignores other relevant perspectives, adequate objections, or the context of where the problems arise. Accordingly, not being “practical in approach, philosophically well grounded, cross disciplinary” or not being performed by “good people” or skilled professionals [112] may bias bioethics. The same goes for using ideal theories to tackle problems in non-ideal context [113] or the lack of specifying principles [114]. This does not make for example an explicitly stated virtue-ethical analysis of euthanasia biased, because the moral theory is explicitly declared. However, if the author uses the outcome from such an analysis to draw general conclusions, one could argue that the work is biased.

Yet another type of bias inherent in moral theories is what may be called *conceptual bias*. For example, it has been argued that there is a basic asymmetry in ethics, making some concepts, such as *bad*, easier to define and grasp than others, like *good* [115]. The same goes for disease versus health [116]. If there are structural asymmetries in moral concepts and ethical theories, this can bias our judgments in bioethics.

Furthermore, it has been pointed out that certain biases are more likely in specific moral theories. For example, it has been argued that there is a potential bias in casuistry, e.g., in describing, framing, selecting and comparing cases and paradigms [117]. The reason is that in order to assess relevance (of a case), we rely on general views, which may be biased. Correspondingly, it has been argued that the use of (constructed) case studies may mislead moral reasoning [118]. According to these lines of thought, it may be possible to assess various kinds of moral theories for their “characteristic biases.”

On the other hand, various types of cognitive biases may distort bioethical reasoning (in many theories). Dupras and colleagues identify three such cognitive biases that may impede ethical reasoning: exceptionalism, reductionism, and essentialism [119] where genetic exceptionalism, genetic reductionism, and genetic essentialism serve as examples.

Another theory type of bias is *bias towards inadequate moral perspectives*, i.e., the tendency to the rely on arguments from an erroneous or inadequate moral theory or perspective or to rationalize of a preferred conclusion by appeal to arguments that underpin a preferred conclusion, which e.g., has been identified in clinical ethics [12]. On the other hand, lack of theoretical foundation (in moral philosophy) [120], lacking specific theoretical foundations (such as utilitarianism + decision theory) [121], or not being principle based [122, 123] may also hamper and bias bioethical analysis, it is argued. Others have pointed out that the lack of “sensitivity to the problem of the multiplicity of moral traditions” [99] could bias bioethics work. While interesting, there may be very many views on what an “inadequate moral perspective” is and difficult to decide what counts as adequate. Nonetheless, there may be some agreement on adequacy, e.g., that ethics of proximity may be less relevant for the assessment of cost-effectiveness than utilitarian calculus.

The point here is that there may be implicit theoretical assumptions that may bias bioethics work. The same seems to be the case for our analyses.

#### Analysis bias

There are also potential biases related to ethical analysis in a broader sense. *Myside bias* is one example of this, according to which we can have a tendency to evaluate or generate evidence, test hypotheses, or analyze or address moral issues in a manner biased toward our own prior perspectives, opinions, attitudes, or positions [124]. At the same time it has been shown that we may consider one-sided arguments to be better than balanced arguments (even if they are opposite to one’s opinion) [125]. The way we assess arguments, weigh the various factors, and synthesize a topic may certainly be biased by unconscious mechanisms.

Moreover, the processes of specifying [126], interpreting [114], or balancing [127, 128] moral norms, values, and/or principles may be biased. Ethical work can also contain “moral fictions” biasing the analysis [129]. Moral fictions have been defined as “false statements endorsed to uphold cherished or entrenched moral positions in the face of conduct that is in tension with these established moral positions” [130]. However, labelling something as a “moral fiction” can itself introduce bias (see *terminology* bias above).

It has also been suggested that we can make “moral errors” or “moral fallacies” [131] due to various biases, such as *psychic numbing*, *the identifiable victim effect*, and *victim-blaming* [132].

Again, implicit assumptions or tendencies in our analyses may bias bioethics work.

#### Argumentation bias

Flawed arguments and fallacies in argumentation can also bias bioethics work. (Most) bioethicists are trained in detecting and avoiding flawed arguments, such as fallacies of vagueness, ambiguity, relevance, and vacuity [133]. However, the reviewed literature identifies flawed moral reasoning [134] or bad arguments that do not fall under the groups of illogical or flawed arguments [135]. Some of these can be characterized as rhetoric, deception, or argumentative techniques. The list of logical fallacies and bad arguments is long [133] and beyond the scope of this article. Here only some examples of how they may bias bioethics work are included to illustrate their profound importance for biasing bioethics work.

*False analogies* can bias arguments if there are morally relevant differences between the case and the analog. One example in bioethics is revealing false analogies in the argument for coercive measures against alcohol consumption during pregnancy, where it has been argued that using court orders to medically treat women (for alcohol dependency) during pregnancy is analogue to coercion by “physically abusive partners” [136].

Moreover, reasoning from *is* to *ought* can bias bioethics work. This is related to what has been called “Hume’s law,” “Hume’s guillotine,” or “the is-ought-fallacy,” and to”the naturalistic fallacy” attributed to George Edward Moore (1873–1958) [137]. It is also related to the reasoning from quantity to quality, e.g., in the enhancement debate where it is argued that more intelligence is better [41].

Accordingly, *inference from description to prescription* is a well-known challenge where ethical conclusions are based on opinion polls [138]. The number of empirical work in bioethics has increased substantially the last decade [139] improving the empirical premises for ethical analyses, but also posing challenges [140]. As knowledge about people’s attitudes towards biotechnologies, such as genetically modified human germlines, are used to inform policy making [141] they may also come to influence ethical analyses and argumentation.

Relatedly, the *experience paradox* is an appeal to experience which represents a “wide-ranging and under-acknowledged challenge for the field of bioethics” according to which personal experience is a liability in bioethics debates when they express vested interests or are not representative of those involved [142]. This relates to epistemic (testimonial) injustice [143].

Related to some of the framing effects that have been discussed above, we may in bioethics use vague, unclear, or ambiguous concepts, which can confuse, obfuscate, or frame the argument in unwarranted ways. One example of this is the use of the concept “naturalness” (for example in the enhancement debate) which has been shown to be used in a number of ways confusing rather than clarifying arguments [144]. Admittedly, vagueness can be beneficial in bioethics [145–147]. However, it can also confuse arguments or stop them, e.g., in statements such as “that is not natural” or “that breaches with personal autonomy."

Related to the *yuck-factor* (see above) bioethicists can also appeal to effects of revulsion, repugnance, abhorrence, or repulsion [148] in their work. While moral disgust may play a role in bioethics, it may also be used in a manipulative way and bias an analysis or argument.

"Begging the question," or *petitio principii*, is the tendency to assume the conclusion of an argument. This form of argument can result in bias in bioethics, for example in debates on the beneficial outcomes of proton therapy for the treatment of cancer, it has been argued that it is unethical to waste time in assessing its outcome by high-quality trials as its outcomes obviously must be beneficial [149]. This relates to *progress bias*, discussed above.

Bias can also result from assuming controversial premises (without justification), drawing conclusions beyond premises, using obscure or controversial examples, analogies, or thought experiments [150], or concluding without assessing the truthfulness or plausibility of crucial premises [16]. The same goes for *straw man arguments* (refuting something else), *argument selection*, and not addressing relevant counterarguments.

While clearly not exhaustive, the examples illustrate that there many ways that flawed arguments can distort biases in bioethics work.

#### Decision bias

Many biases also appear in moral decision-making, and many of them have been mentioned under cognitive and affective biases. While biases in decision-making merit a separate study [30], three main types of decision biases should be mentioned [151].

First, *simplification biases* can be observed when decisions are made based on selected and limited empirical evidence, e.g., when they are insensitive to base rates or are based on illusory correlations—or when only some of the empirical premises are taken into account.

Second, *verification biases* occur when decisions are made to stick to status quo or maintaining consensus in the group, e.g., when decisions are made to maintain consistency in a group and the experience of control.

Third, *regulation biases* are tendencies to avoidance temperaments in ethical debates for example in “rationalizing or downplaying the seriousness of ethical dilemmas and avoiding taking personal responsibility due to feelings of discomfort” [30].

The moral biases are summarized in Table 4.

**Table 4:** Moral biases relevant for bioethics with type of bias, short description, and an indication for what type of bioethics this bias may be most relevant to assess

Type of bias	Bias	Definition/short description	Type of bioethics
Framing	Tinting, coloring	The tendency to give tinted or colored presentations of states of affairs, arguments	A, ER, EA, ELS, CEC, PEC
	Nudging	The tendency to “push” the interlocutor or decision-maker in a specific direction	A, EA, PEC
	Terminology	The tendency to talk about groups in terms of their conditions (“diabetics,” “epileptics” etc.)	A, ER, EA, ELS, CEC, PEC
	Embeddedness	The tendency that basic conceptions are personally, socially, and culturally embedded	A, EA, CEC
	Standpoint adherence	The tendency to stick to one’s standpoint despite strong evidence against it	A, EA, CEC, PEC
	Impartiality illusion	The tendency to think that one’s work is impartial	ER, EA, ELS, PEC
	Expectation bias Conflict of interest	The tendency to provide outcomes in accordance with what is expected Shaping the perception of a situation or task in a subconscious manner	A, ER, EA, ELS, CEC, PEC
	Delimiting effect	Directing the debate by defining what is the issue or what is an ethical question (or not)	A, EA, CEC, PEC
Moral theory bias	Theory dominance	The tendency to let one theoretical perspective dominate the analysis. Ignoring other relevant perspectives (objections or practical problems)	A, EA, CEC, PEC
	Bias towards inadequate moral perspectives	The tendency to rely on arguments from an erroneous or inadequate moral theory or perspective or to rationalize a preferred conclusion by appeal to arguments that underpin a preferred conclusion	A, EA, CEC, PEC
	Conceptual bias	Structural asymmetries in ethics that can influence ethical judgments	A, ER, EA, ELS, CEC, PEC
	Theoretical direction-lessness	Lack of theoretical foundation (in moral philosophy) or lack of well-founded principles	A, ER, ELS, CEC
Analysis bias	Myside bias	The tendency to evaluate or generate evidence, test hypotheses, or analyze or address moral issues in a manner biased toward their own prior perspectives, opinions, attitudes, or positions	A, EA, CEC
	Specification, interpretation, or balancing bias	Bias in the process of specification, interpretation and/or balancing of moral norms, values or principles	A, EA, PEC
	Moral fictions	The tendency to endorse false statements to uphold cherished or entrenched moral positions in the face of conduct that is in tension with these established moral positions	A, EA, CEC, PEC
	Moral errors	“Moral fallacies” due to various biases, such as *psychic numbing*, *the identifiable victim effect*, *victim-blaming* etc	A, EA, CEC, PEC
Argu-mentation bias	False analogy False equivalence	Using an analogy that has morally relevant differences from the analog False analogies are related to making misleading or false comparisons and *availability bias*	A, EA, CEC, PEC
	Inferring from description to prescription	Arguing from empirical facts (about peoples’ opinion) to ethical conclusions	A, ER, EA, CEC
	Using ambiguous concepts	The tendency to use unclear or ambiguous concepts that can confuse or confound the argument	A, ER, EA, ELS, CEC, PEC
	Repulsion and moral disgust	The tendency to appeal to repulsion or moral disgust in order to obtain an argumentative effect	A, CEC
	Straw man argument	Giving the impression of refuting an opponent’s argument by (covertly) replacing it by a different argument	A
	Argument selection	The tendency to select (counter-)arguments that are easy to rebut or to present them in uncharitable ways	A, EA, CEC, PEC
	Argumentum ad hominem	Attacking the character, motive, or attribute of the person making an argument rather than the substance of the argument itself	A
	Relevance fallacy	Presenting a sound argument, which fails to address the issue in question	A
	Red herring	The tendency to divert an argument to unrelated issues (Ignoratio elenchi)	A
	Experience paradox	An appeal to personal experiences in bioethics debates where the experiences express vested interests or are not representative of those involved	A, EA, CEC, PEC
	Petitio principii	The tendency to assume the conclusion of an argument, i.e., "begging the question," which is a kind of circular reasoning	A, EA, CEC, PEC
	Argumentum ad populum	The tendency to assert that everyone (else) agrees (also called “bandwagoning”)	A, CEC
	Either-or fallacy	The tendency to create a false dilemma in which the circumstances are oversimplified (also referred to as “false dichotomy”)	A, CEC
	Card stacking, cherry picking	The tendency to select facts, examples, analogies, thought experiments that fit to one’s conclusion	A, ELS, CEC, PEC
	Double standards	The tendency to demand less from one’s own arguments than from opponents’	A, EA, CEC, PEC
	Hasty generalization	The tendency to “jump to conclusions” or generalizing quickly and/or sloppily	A
	Unclear or mispresented argument	The tendency to present the argument one is attacking or opposing in a misleading manner or in a way that the opponent does not recognize or acknowledge	A
	Controversial premises	The tendency to presume controversial premises without discussing their truthfulness or adequacy	A, EA, PEC
	Implicit theoretical assumptions	The tendency to make implicit crucial theoretical assumptions and definitions and drawing strong conclusions without showing how they rely on these assumptions	A, EA, CEC, PEC
	Conclusion bias	The tendency to draw conclusions beyond the limitations or premises of the argument	A, EA, CEC, PEC
Decision bias	Simplification bias	The tendency to base decisions on illusory correlations and to be insensitivity to base rates	A, EA, ELS, CEC
	Verification bias	The tendency to base decisions on illusions of control and self-serving bias	A, EA, PEC
	Regulation bias	The tendency to make framing errors and regret avoidance	A, EA, CEC

*A* Agitation, *ER* Empirical Research, *EA* Ethical analyzes, *ELS* Ethics literature synthesis, *CEC* Clinical ethics consultation, *PEC* Philosophical, Ethical, and Conceptual analyzes

### Measures to address or avoid bias

As there are many biases there are also many ways to address them. For example, some call for a ‘critical bioethics’ where the ethical analyses in bioethics are empirically rooted [152], others argue for providing a reflexive autoethnographic account of arguments in bioethics applying “confessional tales” [88], and some urge to acknowledge the importance of (framing of) stories [153].

Special (reverse) tests have been suggested to avoid specific biases, such as the status quo bias [46] and progress bias [58]. Adhering to criteria for good ethical argumentation, such as the Rapoport rules (after the Russian-born American game theorist Anatol Rapoport) [154] or the many sets of criteria for “good bioethics” [112, 122, 123, 130, 150, 155–163] may help avoiding the negative effects of biases in bioethics. Correspondingly, declarations of biases together with (or as part of) declarations of conflicts of interest may also reduce (the effects of) biases.

Moreover, in the general literature on biases there are many advice on debiasing [164, 165] and compensatory measures (such as nudging). Such suggestions also exist for health decisions in the clinical setting [31, 166–168]. “Moral debiasing” has also been suggested [169]. Clearly, several of these approaches and attempts may be relevant for bioethics as well. However, to decide which biases should be addressed and in what manner and how to address mistaken moral judgments (or moral heuristics) [170] is another big issue which is beyond the scope of this study. Here the point has been to provide an overview of biases that are relevant to bioethics work, to suggest a way to classify them, and to stimulate reflection, debate, and further research.

## Discussion

This review provides an overview over biases that can be relevant to bioethics work. While most of the biases are well-known from other fields, such as psychology and behavioral economics, their relevance has been investigated in the context of bioethics work and with examples from bioethics. Moreover, the article has suggested a way to classify biases, i.e., in terms of cognitive and affective biases, imperatives, and moral biases that can bias bioethics work. The aim with the overview has been to increase awareness, reflection, and debate on biases in bioethics. The overall and more distal goal is to promote actions towards avoiding or reducing biases, and thereby hopefully to contribute to improve the quality of bioethics.

### Biases by other names

At the outset of this article, I noted that biases have not gained much explicit attention in bioethics. There may certainly be many reasons for this. One reason why we are not that preoccupied with explicitly addressing biases as in other fields can of course be that we do not apply a unified or clearly defined method (that is subject to specific biases) in the first place [171], or that we think that biases are “productive in human understanding” [172] or are shortcuts to effective decisions [173]. Yet another reason may be that bioethics deals with norms and values, which can be thought to introduce “evaluative bias” [174]. Hence, bioethics is inherently biased and haunting biases is counterproductive or even self-destructive. Be that as it may, as I have indicated, there may still be biases that we can and should address and avoid in bioethics.

Another reason for the lack of attention to biases in the bioethics literature can of course be that biases are classified and discussed differently in the bioethics literature. For example, the distinction between withholding versus withdrawing treatment is extensively debated as a philosophical question [175], and not as a bias, such as the *omission bias* (the tendency to judge harmful commissions as worse than equally harmful omissions) [176].

Moreover, bioethicists may be subject to phenomena that are characterized by other words, e.g., “partial moral blindness” [177], “cognitive islands” [178] or to “expert shopping” effects [179]. Moral sensitivity [180], moral judgments, and moral courage [181] may also be interpreted as biases [182]. For example, moral judgments are claimed to be based either on intuitive, default automatic emotional process (deontological) or on conscious, controlled reasoning processes (utilitarian) [183]. Additionally, bioethicists may be subject to misinformation and the spread of false belief [184]. How these phenomena relate to biases is the topic for a separate study.

Yet another reason could be that bioethicists are working with biases in the reasoning and argumentation of important issues, but call them by other names, such as “fallacies” or “flawed argumentation.” Accordingly, we would not need to discuss biases as they are addressed by our methods as such. However, as indicated in this study, it is far from obvious that all biases can be discussed or addressed as philosophical issues. Hence, it still seems important to investigate to what extent biases (as understood and studied in other fields) are relevant to bioethics.

Other areas for reporting, synthesizing, and assessing research results and providing input for decision-making have elaborate systems for assessing bias, such as the ‘Preferred Reporting Items for Systematic Reviews and Meta-Analyses’ (PRISMA) statement [185] and ‘Enhancing transparency in reporting the synthesis of qualitative research’ (ENTREQ). This is much less pronounced in bioethics. While there is some quality assessment in some approaches for literature synthesis in ethics [36], and the upcoming PRISMA Ethics—Reporting Guideline for Systematic Reviews on Ethics Literature mentions the problems of bias (https://osf.io/g5kfb/), there seems to be very limited systematic assessment of bias in ethics. Hence, as long as biases can undermine the quality of research, distort decision-making, and have unjust implications they are ethically relevant. Biases are also demonstrated in ethics in general [30, 39], and it appears reasonable to assume that ethicists are subject to biases as other researchers and persons. In addition to general biases, such as cognitive and affective biases, bioethicists may be subject to specific "moral bias," e.g., related to various types of professional characteristics in our field.

### Psychological versus moral mechanisms

One important objection to this study is that not all biases can be transferred from the field where they are studied (psychology and behavioral economics) to bioethics. Psychological mechanisms may not be relevant for moral phenomena. As already acknowledged, many biases are strongly related to things, while bioethics deal with ideas, norms, values, intuitions, judgments, and arguments. Hence, they may not be of any particular relevance. However, the main line of reasoning in this article has been that: (1) general psychological mechanisms influence bioethicists in the same manner as the general population and may thus be relevant in bioethics work because they can undermine good judgment. (2) the psychological mechanisms described as biases for one type of entities (e.g., decisions on giving away or selling items) may be relevant for the entities or phenomena in another area (e.g., the reluctance to give up ideas, positions, or perspectives in bioethics). (3) providing examples illustrating the relevance of the specific biases for bioethics.

Clearly, I have not provided empirical evidence for these “moral mechanisms” and their relationship to the “psychological mechanisms of bias” [186]. That is beyond the scope of this study and an important topic for further research. However, there clearly exist experiments and studies that measure bias in bioethics [30, 186].

### Biases and bad bioethics

I have taken as a point of departure that biases may distort bioethics work and that identifying and addressing them may improve the quality of our work. I have also briefly said something about potential measures to address or avoid biases. I have left it open for debate and future research to decide on which biases that should be addressed or avoided in what type of bioethics work. The obvious reason is that it would have been beyond the scope of this article. Nonetheless, I hope that the overview and taxonomy provided in this study can stimulate future debates and studies.

One important issue to consider, and which makes this particularly interesting, is that some types of bioethics work may be very biased, and still be of high quality. For example, a study on abortion by a Catholic academic or by a feminist may be biased in many of the ways described in this study, but still be of high quality. Accordingly, Jansen and Sulmasy argue that “good arguments can come from any quarter and that no argument should be dismissed or discounted simply because of its source”[28].

Nonetheless, as revealing conflicts of interests can distort the value of evidence, so can the uncovering of biases undermine the power of an argument. A bias is an indicator of, but not a sufficient condition for poor quality bioethics.

Certainly, there are many things that can undermine good bioethics other than biases [150]. Hence, avoiding biases will not guarantee the quality of bioethics. However, it can contribute to improving quality together with many other measures. While other quality criteria have got quite some attention [112, 122, 123, 130, 155–163], many of the biases identified in this study have not.

Accordingly, we need to qualify the relationship between biases and quality in bioethics. I hope that this study can provide a good starting point for such work.

## Limitations

The topic of this study is extensive and there are certainly many limitations. First and foremost, this is a narrative (overview) review with limitations [37]. It is not exhaustive. The selection of biases included in the study is limited and can itself be criticized for being biased. As stated openly at the outset, there are very many biases identified in the literature. All cannot be included, and admittedly I have made a selection. Thereby, many types of biases may have been excluded. For example, bioethicists are subject to pressure to publish and may be motivated by to pose controversial claims that may make them cited and renown [88]. Moreover, many other specific biases, such as *the bandwagon effect*, can be relevant also in bioethics [20].

Yet another bias that is not included, is the so-called *moral credential effect*, which occurs when someone who does something good gives themselves permission to be less good in the future. Although morally interesting, it may not be that relevant to bioethicists. Yet another topic that is not covered properly is biased decision-making processes [187]. Moreover, many of the common mechanisms and drivers that concern health professionals, researchers, and policy- and decision-makers in general will also apply to bioethicists.

There are also various kinds of biases that can occur due to ethical requirements. One such example is “consent bias” which is a bias in those that enter a research study because the consent process results in bias making the participants not being representative for the general population [188]. Such biases are also beyond the scope of this article although they are ethics-related biases.

Although there are relevant arguments for including moral intuitions into biases [189], this would render this study too comprehensive. Moreover, the relationship between moral intuitions and biases is complex [190, 191] and warrants a separate study.

While admittedly having excluded relevant biases, such as "practitioner bias” [182], it may also be claimed that I have been too inclusive, as I have included the is-ought-fallacy, which is a form of invalid deductive argument. Including invalid arguments (in propositional logic) would arguably be to stretch the term bias too far. I fully agree that invalid arguments in bioethics should be addressed with the terminology and methodology of logic and argumentation theory. I have rather included some examples of how flawed arguments can bias bioethics. Formal, informal, inductive, and other fallacies as well as flaws in moral reasoning are dealt with in more length and detail elsewhere [133, 134].

There are also crucial philosophical questions following from the various types of biases that have been presented and (partly) discussed here, for example what our responsibility for the biases are [192]. While such issues are important, they could not be included in this study.

Another important caveat is that the various biases do not apply all the time or in all contexts. While I have tried to indicate in which type of bioethics work they may be most relevant, their relevance may vary from country to country and from topic to topic. For example, conflict of interests due to stakeholder expectations may vary from place to place.

One profound objection, that has already been mentioned, is that biases are basic to human beings and that it is impossible to eliminate biases [20, 193]. Accordingly, unbiased bioethics is an utopian aim that should be abandoned [172]. As already admitted, deciding whether biases can be eliminated, reduced, or mitigated and how is beyond the scope of this study. However, implicit in the study lies a belief (a bias) that identifying biases and increasing the awareness of them can be helpful for bioethics work. For example, one area where it can be helpful is in the appraisal of quality of normative literature [194]. Even if all biases may not be eliminable, some may very well be, while others may be reducible. Hence, there are good reasons to acknowledge and address biases in order to promote transparent and disciplined ethical work.

There are several challenges with revealing biases. First, the baring of biases may itself be biased. Second, revealing biases may require knowledge about the bioethicist, e.g., with respect to perspective, position, inherent inclinations etc., which we may not have access to. Third, there may be disputes about biases as all other things in bioethics. Hence, while revealing, measuring, and assessing biases may be difficult, awareness and open discussions on biases may still be valuable and improve the quality of bioethics work.

As the identification, assessment, explanation, and classification of biases may itself be subject to biases, we may have higher order biases. As this study presents an overview of biases, it is open for others to analyze, assess, and criticize them. The study does not provide evidence of the prevalence and impact of the biases, which invites to further research.

Correspondingly, the various types of biases (cognitive, affective, imperatives, and moral biases) are not natural kinds, and the borders between them are blurred. Admittedly, the same goes for the grouping of biases. As shown, the various types of biases are also interconnected. Hence, the classification is open for discussion. The same is the indication of how relevant the biases are for the various kinds of bioethics work, as already pointed out.

Moreover, there are many relationships between psychological biases, imperatives, and moral biases. For example, biases here classified as moral biases, such as the framing effects, analysis bias, and decision bias are rooted in or related to corresponding cognitive biases. Correspondingly, there are relationships between various cognitive biases, such as the *status quo bias* and the *endowment effect* and between *progress bias* and *pro-innovation bias*, as already mentioned.

Additionally, the term “moral bias” is by no means new. It has been mentioned in the literature before [191] and relates to terms like “moral sentiment,” “moral distortion,” “moral blindness,” “moral dogmatism” [195], “moral prejudice,” and “moral intuition” [189], as already mentioned. Moreover, moral biases may be interpreted in many ways, e.g., in terms of moral heuristics. According to Gerd Gigerenzer our moral decisions are fast and frugal and do not depend on our preferences or deliberate reasoning [189]. The issue of moral heuristics is a topic of its own merits. The point here has not been to enter the general debates on moral bias, but only to use the term to refer to a class of biases that can distort bioethics work.

Another interesting topic for further research is the difference between biases and doing wrong. For example, it could be argued that doing moral theory wrong is not a bias, it is just being a poor bioethicist. Both such errors and biases are unconscious, and both may be recognized by the person having or doing them. However, errors appear to be more easily acknowledged and corrected than biases. In any case, the issue deserves more attention than given here.

Yet another issue that is beyond the scope of this article, but which is highly relevant for further research, is whether biases in bioethics can be measured. In the field of evidence production there are many approaches to classify and measure biases, and one could envision that such approaches could be established in bioethics as well in order to make proper quality assessment of bioethics work. While measuring biases is not well developed in bioethics, data programs with syntactic and semantic tests for detecting bias have been suggested and demonstrated [196]. Moreover, a self-report measure that assesses an individual's propensity to express specific types of decision biases in ethics, i.e., the Biased Attitudes Scale (BiAS), has been validated [30]. This still is in its infancy and needs further development and future research.

## Conclusion

A wide range of biases have been identified as relevant for bioethics work: cognitive biases, affective biases, imperatives, and moral biases. In particular, moral biases have been discussed in terms of (1) Framings, (2) Moral theory bias, (3) Analysis bias, (4) Argumentation bias, and (5) Decision bias. Based on the general literature on bias and the bioethics literature, specific biases with relevance to various types of bioethics work have been recognized, classified, and explained. Selected examples are provided to help identify and address them.

There are two important reasons to identify and address biases in bioethics. First, analyzing biases in bioethics can help us assess the quality of bioethics work. Second, and consequently, identifying and addressing biases can be used to improve the quality of our work.

Accordingly, the provided overview can be useful to draw attention to biases, as awareness is the first step to addressing and handling biases in bioethics. The overview can also be developed into more formal bias checklists and risk of bias assessments. However, this needs careful deliberation, as there is no simple relationship between biases to quality. Nonetheless, I hope that this overview can be a helpful starting point for further research that will be valuable for bioethics work.

## Data Availability

All data are available in publication.
